# Genomic epidemiology of a protracted hospital outbreak caused by multidrug-resistant *Acinetobacter baumannii* in Birmingham, England

**DOI:** 10.1186/s13073-014-0070-x

**Published:** 2014-11-20

**Authors:** Mihail R Halachev, Jacqueline Z-M Chan, Chrystala I Constantinidou, Nicola Cumley, Craig Bradley, Matthew Smith-Banks, Beryl Oppenheim, Mark J Pallen

**Affiliations:** Institute of Microbiology and Infection, University of Birmingham, Birmingham, B15 2TT UK; Division of Microbiology and Infection, Warwick Medical School, University of Warwick, Warwick, CV4 7AL UK; NIHR Surgical Reconstruction and Microbiology Research Centre, Queen Elizabeth Medical Centre, Birmingham, B15 2TH UK

## Abstract

**Background:**

Multidrug-resistant *Acinetobacter baumannii* commonly causes hospital outbreaks. However, within an outbreak, it can be difficult to identify the routes of cross-infection rapidly and accurately enough to inform infection control. Here, we describe a protracted hospital outbreak of multidrug-resistant *A. baumannii*, in which whole-genome sequencing (WGS) was used to obtain a high-resolution view of the relationships between isolates.

**Methods:**

To delineate and investigate the outbreak, we attempted to genome-sequence 114 isolates that had been assigned to the *A. baumannii* complex by the Vitek2 system and obtained informative draft genome sequences from 102 of them. Genomes were mapped against an outbreak reference sequence to identify single nucleotide variants (SNVs).

**Results:**

We found that the pulsotype 27 outbreak strain was distinct from all other genome-sequenced strains. Seventy-four isolates from 49 patients could be assigned to the pulsotype 27 outbreak on the basis of genomic similarity, while WGS allowed 18 isolates to be ruled out of the outbreak. Among the pulsotype 27 outbreak isolates, we identified 31 SNVs and seven major genotypic clusters. In two patients, we documented within-host diversity, including mixtures of unrelated strains and within-strain clouds of SNV diversity. By combining WGS and epidemiological data, we reconstructed potential transmission events that linked all but 10 of the patients and confirmed links between clinical and environmental isolates. Identification of a contaminated bed and a burns theatre as sources of transmission led to enhanced environmental decontamination procedures.

**Conclusions:**

WGS is now poised to make an impact on hospital infection prevention and control, delivering cost-effective identification of routes of infection within a clinically relevant timeframe and allowing infection control teams to track, and even prevent, the spread of drug-resistant hospital pathogens.

## Background

*Acinetobacter baumannii* is an important cause of nosocomial infection, particularly ventilator-associated pneumonia and bloodstream infections in critically ill patients, and has a tendency to cause hospital outbreaks [1,2]. Multidrug-resistant (MDR) and even pan-drug-resistant strains have been reported worldwide [3]. It has also emerged as a threat to casualties of the conflicts in Iraq and Afghanistan, with the secondary problem that strains introduced to hospitals by military personnel can cause cross infection of staff and patients [4-9]. Although existing molecular typing methods play an important role in identifying outbreaks [10,11], they lack the resolution necessary to identify chains and modes of transmission within outbreaks and so can provide only limited guidance to infection control teams on how best to control or terminate an outbreak.

Whole-genome sequencing (WGS) of bacterial isolates provides a promising new method for investigating the epidemiology of outbreaks, particularly when coupled to clinical locational and temporal data [12-17]. Here, we describe a protracted hospital outbreak which occurred in Birmingham, England between July 2011 and February 2013 and was caused by a strain of *Acinetobacter baumannii* belonging to pulse-field gel electrophoresis type (pulsotype) 27. During the outbreak, we used genome sequencing to obtain a high-resolution view of the relationships between isolates, allowing us to reconstruct chains of transmission, confirm or refute epidemiological hypotheses and to provide the infection control team with useful insights into the sources and routes of infection during this outbreak.

## Methods

### Microbiological investigations

Here, we report a routine and clinically indicated infection control investigation into an outbreak, with no experimentation on human subjects. No additional samples other than those that were clinically relevant were taken from patients and the use of genome sequencing falls under the remit of laboratory method development, which does not need ethical approval. Multidrug-resistant *Acinetobacter* (MDR-Aci) isolates were obtained from routine clinical samples through culture on blood agar, followed by single-colony isolation. Bacterial identification and antibiotic susceptibility testing were performed in the hospital microbiology laboratory on the Vitek 2 system according to the manufacturer’s recommendations (bioMérieux, Basingstoke, UK) [18]. Multidrug resistance was defined as resistance to ≥3 classes of antibiotics (quinolones, extended-spectrum cephalosporins, β-lactam/β-lactamase inhibitor combinations, aminoglycosides and carbapenems).

All MDR-Aci isolates from the Queen Elizabeth Hospital Birmingham during the outbreak period (July 2011 to February 2013) were considered for inclusion in the study. During this period, 65 patients tested positive for MDR-Aci in the clinical laboratory. Patients were numbered consecutively, based on the date of first isolation of MDR-Aci. The initial MDR-Aci isolate from each patient was sent to the Laboratory of HealthCare Associated Infection in Colindale, London for speciation and typing by pulsed-field gel electrophoresis (PFGE) and other molecular methods [10]. When the reference laboratory finds that two or more isolates from the UK share a novel PFGE pattern, the isolates are assigned to a new numerical pulsotype, for example, pulsotype 27 or pulsotype 29.

An attempt was made to propagate isolates from all MDR-Aci-positive patients for genomic analysis. However, isolates from three patients (patients 15, 28 and 38) were lost on sub-culture or contaminated, leaving us with 74 genome-sequenced pulsotype 27 isolates from 58 patients. To examine within-host diversity, multiple isolates were obtained from 13 patients from different body sites and/or at different times. In addition, 18 isolates from 15 patients that had been identified as *A. baumannii* complex by Vitek 2, but turned out not to belong to the outbreak, were subjected to genome analysis, as were 10 environmental isolates and four control strains, which had been subjected to prolonged subculture in the laboratory. We also genome-sequenced the first pulsotype 27 isolate from the UK (kindly supplied by Jane Turton at the Laboratory of HealthCare Associated Infection), which was recovered in 2006 from a patient that had recently undergone surgery in India.

### Genomic and epidemiological investigation

Genomic DNA was extracted from 114 putative *Acinetobacter* isolates, applying Qiagen 100/G Genomic-tips to 5 to 10 mL of overnight culture. A barcoded fragment library was generated for each isolate using the Nextera Sample Preparation and Nextera Index Kits (Illumina), then sequenced on an Illumina MiSeq, using paired-end (2 × 151 or 2 × 251) protocols, to give a minimum depth of coverage of 10×. We implemented a filtering pipeline that trimmed reads at both ends, removing adaptors and bases with sequencing quality <20, and discarded all reads that mapped to PhiX or that contained Ns or where >20% of bases had a sequencing quality of <20.

The genome of an isolate from a patient early in the outbreak (patient 6) was sequenced on two different sequencing platforms (454 FLX + and Illumina MiSeq), then a hybrid assembly was created to provide a reference genome for the outbreak, using Newbler v2.6 [19]. This assembly consisted of 4,031,405 base pairs, with 160 contigs in total and 126 contigs >500 bp, with an N50 for contigs >500 base pairs of 31,936 base pairs. Five contigs (seq23, 67, 75, 100 and 128), comprising 77,648 base pairs/80 CDSs, were assigned to a cryptic plasmid on the basis of read depth, patterns of absence in some isolates and homology searches.

The outbreak reference genome was compared to all the MDR-Aci genome sequences that were publically available in May 2013, using the Average Nucleotide Identity (ANI) approach to identify the closest genome-sequenced strain [20,21]. Isolates were assigned to a species on the basis of ANI to reference genomes [20,21]. For genotypic investigations of potential outbreaks, genome sequences were mapped to the relevant reference genome using Bowtie 2 [22], with default parameters, except that the reads were soft-clipped at the ends to improve the alignment score (option --local).

#### SNV discovery procedure

After mapping each set of read data to the reference genome as explained above, we processed with SAMtools v0.1.18 [23] (*mpileup* with default parameters, disabling the probabilistic realignment for the computation of base alignment quality, that is, we used option -B) and filtered it using BCFtools v0.1.17-dev (using the *vcfutils.pl varFilter* script to find variants with minimum root-mean-square mapping quality of 30, maximum read depth of 10,000 and minimum distance to a gap of 150 bp, that is, approximately one read length). Using custom scripts, we screened these SNV locations to exclude some potentially spurious SNVs by retaining only SNVs which are:

- not from SNV-dense regions - no more than three SNVs in a 1,001 bp window centred on the SNV location
- most likely not from repeat regions – coverage less than twice the average isolate’s coverage and
- at least 150 bp from scaffold boundaries.

The alignments of the remaining variant loci were then manually inspected to check quality. For all SNV loci with coverage five-fold or less or with consensus <90%, we performed PCR and Sanger sequencing as a SNV verification step.

For isolates from the pulsotype 27 outbreak, we defined a new major SNV genotype, with a numerical designation (1.0, 2.0 and so on) whenever isolates with the same SNV profile were obtained from more than one patient. Genotypes confined to single patients within the outbreak were defined as minor genotypes and were given serial numerical designations (1.1, 2.1, 2.2 and so on) based on the closest relationship to a major genotype. All sequences from this study are available under ENA Accession number PRJEB4735.

To reconstruct the most parsimonious set of transmission events, we used a custom-built algorithm and script. We considered three modes of transmission: direct ward contact, where donor and recipient are on the same ward at the same time; delayed ward contact, where donor and recipient have been on the same ward but not at the same time; and theatre contact, where donor and recipient have received treatment in the same operating theatre. In the latter two modes, we assumed that MDR-Aci strains could survive in the hospital environment for up to 5 weeks [24].

## Results

### Description of the *Acinetobacter baumannii* pulsotype 27 outbreak and the outbreak strain

Queen Elizabeth Hospital Birmingham (QEHB) is a public hospital with around 1,200 beds. British military casualties are usually repatriated here. In July 2011, we saw the first case in a new MDR-Aci outbreak: a military patient, who had been admitted to the hospital after suffering a blast injury in Afghanistan. The outbreak strain was identified in the clinical laboratory as an MDR-Aci showing resistance to multiple classes of antimicrobial agent, including aminoglycosides, fluoroquinolones, carbapenems, cephalosporins, carboxypenicillins and tetracycline. It was variably sensitive to colistin. The reference laboratory identified it as *A. baumannii* and, using PFGE, assigned it to pulsotype 27, a pulsotype not previously seen at QEHB.

**Figure 1:**
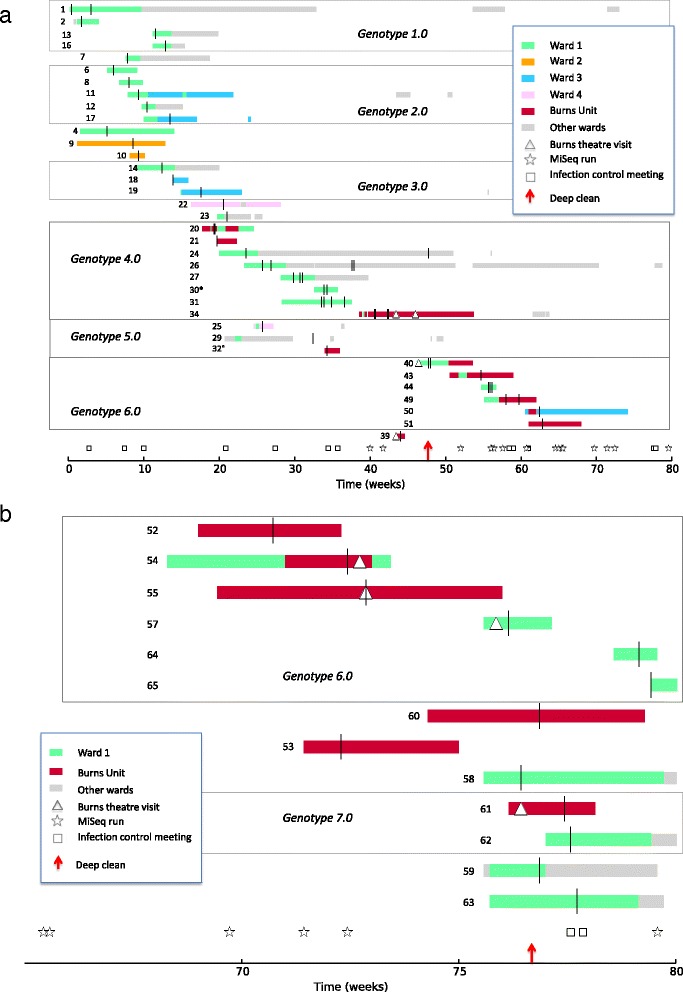
**Chronology of the** ***Acinetobacter baumannii*** **pulsotype 27 outbreak in Birmingham, UK, 2011 to 2013, showing ward occupancy and other events for 52 patients. (a)** The first phase of the outbreak, up to week 70. **(b)** A detailed view of the second phase of the outbreak, after week 70. Vertical bars indicate samples positive for MDR-Aci. The coloured horizontal bars indicate ward occupancy by patients carrying MDR-Aci. Patients are ordered by the SNV genotype of their MDR-Aci isolates, with major genotypes delineated by rectangles. Ward 1 cares mainly for burns and trauma patients; Ward 2 cares mainly for cardiac surgery patients, Ward 3 cares mainly for trauma patients; Ward 4 for plastic, ear-nose-and-throat, maxillofacial, trauma patients. * The first of three isolates obtained from patient 30 was not genome-sequenced. ° Patient 32 visited Ward 1 for 12 hours.

Over the following 80 weeks, isolates of the outbreak strain were recovered from an additional 51 patients, including civilians and military casualties, spanning multiple wards (Figure 1). Isolates were assigned to the outbreak provisionally on the basis of antibiogram and definitively on the basis of pulsotype. However, after we began to apply WGS to putative MDR-Aci isolates in week 40 of the outbreak (April 2012), we also used genome sequence information to determine which isolates belonged to the outbreak. Analysis of a reference genome for the outbreak, created from an early outbreak isolate (isolate 6), revealed that the outbreak strain was distinct from all other well-characterised strains, including strains from previous outbreaks in local hospitals and clusters with other strains in a previously defined, widespread clonal lineage, International Clone I. A comparison with the first British pulsotype 27 isolate, which had been identified by the Laboratory of HealthCare Associated Infection in 2006, showed over 1,200 SNVs, ruling out any close relationship between our 2011 to 2013 outbreak and this historical isolate.

To delineate and investigate the outbreak, we attempted to genome-sequence 114 isolates identified as *A. baumannii* complex by the Vitek 2 system and obtained informative draft genome sequences from 102 of them. Seventy-four clinical isolates, obtained from 52 patients (including the index case), and 10 environmental isolates showed sufficient genomic similarity to the outbreak reference strain (≤8 SNVs different) to be ruled into the outbreak (Table 1).

**Table 1:** **Description of 52 patients and 84 isolates associated with the** ***Acinetobacter baumannii*** **pulsotype 27 outbreak in Birmingham, England, 2011 to 2013**

**Patient no. or environmental source**	**Length of hospital stay (days)**	**Isolate no.**	**Time of isolation (days)**		**Genotype**	**SNVs/plasmid loss** **(p indicates loss of plasmid)**
			**From admission**	**From start of outbreak**		
1	231	1a	3	3	1.0	0
		1b	21	21	1.1	1,p
2	24	2	7	12	1.0	0
4	88	4	25	36	2.1	2,4,p
6	29	6	6	42	2.0	2
7	422	7	3	55	1.2	3
8	23	8	9	56	2.0	2
9	83	9	52	60	2.2	2,7
10	15	10	65	65	2.3	2,7,10,11
11	99	11	11	65	2.0	2
12	39	12	6	73	2.0	2
13	62	13	3	81	1.0	0
14	77	14	24	87	3.0	2,6
15	LOST: not included in transmission analysis					
16	31	16	12	90	1.0	0
17	535	17	24	94	2.0	2
18	15	18	2	97	3.0	2,6
19	58	19	19	123	3.0	2,6
20	49	20a	12	135	4.0	2,5,9
		20b	13	136	4.0	2,5,9
21	19	21	1	138	4.0	2,5,9
22	84	22	31	144	2.4	2,5
23	45	23	10	147	2.5	2,5,8
24	218	24a	25	165	4.0	2,5,9
		24b	194	334	4.10	2,5,9,12,p
25	19	25	8	180	5.0	2,5,9,p
26	197	26a	17	180	4.1	2,5,9,13
		26b	25	188	4.0	2,5,9
		26c	100	263	4.2	2,5,9,13,22,p
		26d	100	263	4.2	2,5,9,13,22,p
		26e	102	265	4.6	2,5,9,14
		26f	102	265	4.2	2,5,9,13,22,p
27	82	27a	13	209	4.0	2,5,9
		27b	19	215	4.3	2,5,9,15
		27c	21	217	4.0	2,5,9
28	114	28a	31	227	LOST: mixed culture	
		28b	43	239		
		28c	61	257		
29	64	29*	83	227 (GP)	5.0	2,5,9,p
30	23	30a	10	237	4.0	2,5,9
		30b	13	240	4.0	2,5,9
31	66	31a	37	235	4.0	2,5,9
		31b	37	235	MIXED	
		31c	39	237	4.4	2,5,9,18
		31d	39	237	LOST: mixed culture	
		31e	39	237	*Escherichia coli*	
		31f	39	237	LOST: mixed culture	
		31g	39	237	LOST: mixed culture	
		31h	39	237	LOST: mixed culture	
		31i	46	244	4.5	2,5,9,17
		31j	58	256	4.0	2,5,9
		31k	64	297	*Pseudomonas aeruginosa*	
32	16	32	4	240	5.0	2,5,9,p
34	107	34a	14	284	4.7	2,5,9,19
		34b	15	285	4.0	2,5,9
		34c	15	285	4.0	2,5,9
		34d	15	285	4.0	2,5,9
		34e	26	296	4.8	2,5,9,20
		34f	27	297	4.9	2,5,9,28
38	96	38	39	298	LOST	
39	9	39	4	308	6.1	2,5,9,16,23
40	53	40a	11	334	6.0	2,5,9,16
		40b	13	336	6.0	2,5,9,16
43	60	43	29	383	6.0	2,5,9,16
44	15	44a	8	390	6.0	2,5,9,16
		44b	9	391	6.0	2,5,9,16
		44c	11	391	6.2	2,5,9,16,24
		44e	11	393	6.0	2,5,9,16
		44f	11	393	6.0	2,5,9,16
Ward 1 post-patient 44		E1		397	6.0	2,5,9,16
49	49	49a	21	406	6.0	2,5,9,16
		49b	33	418	5.0	2,5,9,p
50	50	50	14	437	6.0	2,5,9,16
51	96	51	14	440	6.0	2,5,9,16
52	24	52	13	495	6.0	2,5,9,16
53	26	53	6	506	4.11	2,5,9,21,p
54	37	54	30	507	6.0	2,5,9,16
55	47	55	25	510	6.0	2,5,9,16
Burns Unit shower head post-patient 55		E2		532	5.0	2,5,9,p
Burns Unit shower chair post-patient 55		E3		532	5.0	2,5,9,p
Burns Unit patient chair post-patient 55		E4		532	5.0	2,5,9,p
57	12	57	5	533	6.0	2,5,9,16
Touch screen burns theatre post-patient 57		E5		538	7.0	2,5,9,16,26,29
Anaesthetic machine burns theatre post-patient 57		E6		538	7.0	2,5,9,16,26,29
Pat Slide burns theatre post-patient 57		E7		538	6.4	2,5,9,16,26
Stool burns theatre post-patient 57		E8		538	6.4	2,5,9,16,26
Scissors burns theatre post-patient 57		E9		538	6.5	2,5,9,16,27
ECG leads burns theatre post-patient 57		E10		538	7.0	2,5,9,16,26,29
58	72	58	6	535	6.3	2,5,9,16,25
59	29	59	9	538	7.1	2,5,9,16,26,29,30,p
60	36	60	19	538	5.0	2,5,9,p
61	15	61	9	542	7.0	2,5,9,16,26,29
62	27	62	4	543	7.0	2,5,9,16,26,29
63	29	63	15	544	7.2	2,5,9,16,26,29,31
64	8	64	4	554	6.0	2,5,9,16
65	15	65	2	556	6.0	2,5,9,16

Patients were assigned to the outbreak if an initial isolate was shown by PFGE to belong to pulsotype 27. For three patients (15, 28, 38), no MDR-Aci isolates were available for genome sequencing.

*Isolate 29 was obtained after discharge from hospital from a sample provided by a general practitioner (GP).

### Genomics reveals clusters of infection

**Figure 2:**
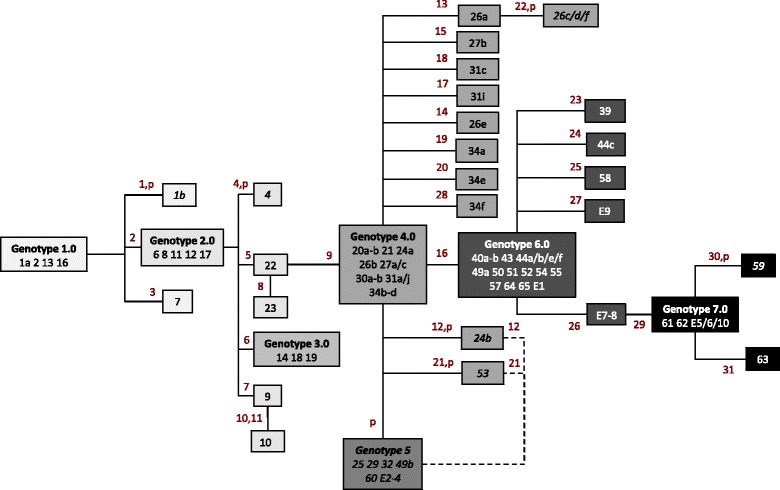
**Genotypes obtained from 84 isolates from the** ***Acinetobacter baumannii*** **pulsotype 27 outbreak in Birmingham, UK, 2011 to 2013, including 74 clinical isolates from 49 patients and 10 environmental isolates.** Numbers in red represent SNVs; ‘p’ indicates loss of plasmid; isolates in italics are plasmid-negative; dotted lines indicate alternative phylogenetic links (plasmid loss then SNV acquisition versus SNV acquisition then plasmid loss).

We identified 31 SNVs in outbreak isolates (Table 2). These SNVs, together with presence/absence of a cryptic plasmid (that is, a 77-kb plasmid with no obvious phenotype), define seven major outbreak genotypes, which fall into a phylogenetic relationship consistent with the timeline of the outbreak (Figure 2). Most of the major genotypes in the outbreak are accompanied by a cloud of one or two SNV variants in a wheel-and-spokes configuration, so that in total there are 32 distinct genotypes of the outbreak strain (Table 1, Figure 2). Laboratory subculture controls, including two different colonies picked after four serial subcultures and a culture that had been subjected to seven freeze-thaw cycles, all had the same SNV genotype as a minimally passaged parent culture, suggesting that SNVs are not readily acquired in the laboratory.

**Table 2:** **Genomic locations and other details of 31 single nucleotide variants (SNVs) detected in the genomes of isolates from the** ***Acinetobacter baumannii*** **pulsotype 27 outbreak in Birmingham, UK, 2011 to 2013**

**SNV no.**	**Location in reference assembly**	**Orthologue annotation**	**Amino acid**		**Codon (residue in bold)**		**Orthologue**
			**Original**	**New**	**Original**	**New**	
1	2354692	**Two-component sensor kinase transcription regulator protein PmrB**	Pro	Leu	C**C**A	C**T**A	AB57_3172
2	1696968	Diguanylate cyclase	Lys	STOP	**A**AA	**T**AA	AB57_0627
3	219628	16S rRNA methyltransferase GidB	Arg	Ser	**C**GT	**A**GT	AB57_1794
4	2953356	3-oxoacyl-ACP reductase	Leu	Trp	T**T**G	T**G**G	AB57_0871
5	2354857	**Two-component sensor kinase transcription regulator protein PmrB**	Thr	Ile	A**C**T	A**T**T	AB57_3172
6	164435	**Adenylate/guanylate cyclase**	Asp	Gly	G**A**C	G**G**C	AB57_1850
7	164513	**Adenylate/guanylate cyclase**	Tyr	Phe	T**A**T	T**T**T	AB57_1850
8	2354642	**Two-component sensor kinase transcription regulator protein PmrB**	Thr	Pro	**A**CC	**C**CC	AB57_3172
9	2568699	Threonine synthase	Leu	Leu	**T**TA	**C**TA	AB57_0327
10	555356	Catalase/peroxidase HPI	Leu	Ile	**T**TA	**A**TA	AB57_0488
11	2961444	AraC family transcriptional regulator	Val	Val	GT**C**	GT**T**	AB57_1179
12	48566	LysR family transcriptional regulator	Leu	Ile	**C**TC	**A**TC	AB57_1964
13	1778342	Lysine/ornithine N-monooxygenase BasC	Trp	STOP	T**G**G	T**A**G	A1S_2384
14	1600195	Bifunctional cyclohexadienyl dehydrogenase/ 3-phosphoshikimate 1-carboxyvinyltransferase	Gly	Ser	**G**GT	**A**GT	AB57_2630
15	3658279	Non-coding	Intergenic 88 bp from start of *serB*				
16	2448345	Plasmid replicase protein	His	Tyr	**C**AC	**T**AC	ACINIS123_A0022
17	706757	Putative transport protein	Ala	Thr	**G**CT	**A**CT	ABAYE2100
18	3286974	ABC transporter ATP-binding protein	Gly	Cys	**G**GT	**T**GT	ABAYE2100
19	2501364	Regulatory helix-turn-helix protein, lysR family protein	Val	Ile	**G**TA	**A**TA	ABBFA_001413
20	2354659	**Two-component sensor kinase transcription regulator protein PmrB**	Arg	Leu	C**G**C	C**T**C	AB57_3172
21	2720233	Non-coding	intergenic 72 bp from start of *kdsD*				
22	3818799	Argininosuccinate synthase	Val	Val	GT**T**	GT**A**	AB57_1152
23	727482	Oxidoreductase short-chain dehydrogenase/reductase family	Leu	Leu	CT**A**	CT**T**	AB57_2417
24	2153319	Diguanylate cyclase/phosphodiesterase	Tyr	Asn	**T**AC	**A**AC	AB57_2291
25	2879522	Glutathionylspermidine synthase	Asp	Glu	GA**T**	GA**G**	HMPREF0022_00853
26	2055876	D-ala-D-ala-carboxypeptidase, penicillin-binding protein	Thr	Lys	A**C**G	A**A**G	AB57_2923
27	2698063	D-and L-methionine ABC transporter ATP-binding protein MetN	Arg	Trp	**C**GG	**T**GG	AB57_1716
28	1499950	Peptidase M20D, amidohydrolase	Glu	Gly	G**A**A	G**G**A	AB57_2996
29	396513	Non-coding	intergenic 80 bp from start of TetR/AcrR transcriptional regulators				
30	2371782	Hypothetical protein	Val	Ala	G**T**T	G**C**T	ACIN5074_3260
31	1935255	Hypothetical protein	Arg	His	C**G**T	C**A**T	AB57_1009

Orthologue designations are taken from the completed genome of *Acinetobacter baumannii* AB0057 (GenBank Accession CP001182). Coding sequences in which more than one SNV occurs are highlighted in bold.

Among the outbreak genomes, we found two protein-coding genes that contained more than one SNV. Four non-synonymous SNVs were identified in the same sensor kinase gene, *pmrB*, which has been implicated in colistin resistance [25]. None of these SNVs match known colistin resistance-associated mutations. However, three of the changes occur in isolates (1b, 34e and 23) that show decreased susceptibility to colistin (MIC 256 mg/L), suggesting that they might represent new resistance-associated mutations. Isolates that share the fourth SNV in *pmrB*, which delineates patient 22 from Genotype 2.0 and also occurs in Genotypes 3.0-7.0, retain sensitivity to colistin (MIC ≤8 mg/L).

### Non-outbreak isolates, within-host diversity and mixed infections

Genome sequencing revealed that 18 *Acinetobacter* isolates, although obtained from hospitalised patients during the study period, did not belong to the main outbreak. A close genomic relationship between four isolates, twinned with a 3-week overlap in ward occupancy, provided evidence of a second small outbreak of MDR-Aci, which from PFGE results on three of the isolates could be assigned to pulsotype 29 (Table 3). Similarly, genome comparisons established that seven isolates obtained from five patients were members of the related species, *Acinetobacter pittii*, but very large pair-wise differences (>20,000 SNVs) between *A. pittii* isolates from different patients ruled out cross-infection.

**Table 3:** ***Acinetobacter*** **isolates from the Queen Elizabeth Hospital, Birmingham, England cultured between July 2011 and February 2013 that do not belong to** ***Acinetobacter baumannii*** **pulsotype 27**

**Patient no.**	**Length of hospital stay (days)**	**Isolate no.**	**Time of isolation (days)**		**Species**	**Pulsotype**	**SNV genotype**
			**After admission**	**From start of outbreak**			
3	72	3	21	13	*A. baumannii*	3	Unrelated
5	22	5	2	27	*A. baumannii*	Unique	Unrelated
26	197	26 g	102	265	*A. baumannii*	Not typed	Unrelated
37	25	37	3	291	*A. baumannii*	Not typed	Unrelated
41	72	41	10	371	*A. baumannii*	13	Unrelated
45	35	45	57	394	*A. baumannii*	Unique	Unrelated
47	48	47	13	401	*A. baumannii*	29	Related
48	167	48	10	404	*A. baumannii*	29	Related
46	33	46a	5	396	*A. baumannii*	29	Related
		46b	7	398	*A. baumannii*	Not typed	Related
56	21	56	18	531	*A. baumannii*	9	Unrelated
33	63	33	9	271	*A. pittii*	Not typed	Unrelated
35	205	35	1	286	*A. pittii*	Not typed	Unrelated
36	27	36	1	286	*A. pittii*	Not typed	Unrelated
42	72	42a, b, c	1	373	*A. pittii*	Not typed	Unrelated
44	15	44d	9	391	*A. pittii*	Not typed	Unrelated

From one trauma patient (patient 26), who was hospitalised for over 7 months, we genome-sequenced seven isolates of MDR-Aci obtained from different anatomical sites over a 4-month period and found five SNV variants (Figure 2):

- The initial isolate, 26a, which was obtained from a sputum sample, falls one SNV away from Genotype 4.0.
- A blood isolate (26b) taken 8 days later falls within Genotype 4.0.
- Isolates 26c/d/f, obtained from a series of CSF samples taken approximately 3 months later, fall one SNV away from 26a
- A second sputum isolate (26e) represents a unique one-SNV variant of genotype 4.0.

Retrieval of a cloud of genotypes from a single patient illustrates the potential for within-host evolution in MDR-Aci, mirroring findings with other hospital pathogens such as *Staphylococcus aureus* [26,27].

From yet another CSF sample from patient 26, we isolated a strain of MDR-Aci that was shown to be distinct from the outbreak strain by PFGE typing and by genome sequencing, providing evidence of double infection. We also found evidence of double infection with *Acinetobacter* in another trauma patient, patient 44, where two isolates, each from a separate wound swab taken on the same day, were identified by genome sequencing as *A. pittii* and the outbreak strain of *A. baumannii.*

### Routes and chains of transmission within the main MDR-Aci outbreak

We reconstructed transmission events, assuming the most parsimonious transmission paths between patients. Using conventional epidemiological information alone, we identified 273 potential transmission events - an average of approximately five per patient - that might link patients within the outbreak. When genome sequence data were included, we were able to reduce this to a set of 57 potential transmission events. This set linked all but 10 of the pulsotype 27 patients and, in most cases, provided a single most-parsimonious transmission event that explained how a patient acquired the outbreak strain (Table 4).

**Table 4:** **Potential transmission events within the** ***Acinetobacter baumannii*** **pulsotype 27 outbreak in Birmingham, England, 2011 to 2013, reconstructed using a parsimonious analysis of ward/theatre occupancy and SNV genotype**

**Patient no.**	**Predicted donor(s) of infection**	**SNVs compared to predicted donor(s)**	**Days between donor and recipient(s)**
1	Index case: a military patient repatriated from Afghanistan		
2	1	0	0
4	1	3	0
6	1	1	0
7	1	1	0
8	6	0	0
9	Unknown		
10	9	2	0
11	6 or 8	0,0	0
12	6 or 8 or 11	0,0,0	0
13	1	0	<14
14	6 or 8 or 11 or 12	1,1,1,1	0
15	Not included in transmission analysis: strain lost on subculture		
16	13	0	0
17	11 or 12	0,0	0
18	11 or 17	1,1	0
19	18 or 11 or 17	0,1,1	0
20	11	2	<21
21	20	0	0
22	Unknown		
23	11	2	<35
24	20	0	0
25	22 or 24	2,1(loss of plasmid)	
26	20 or 24	0,0	0
27	26	0	0
28	Not included in transmission analysis: mixed culture		
29	20 or 24	1,1(loss of plasmid)	0
30	27	0	0
31	27 or 30	0,0	0
32	30 or 31	1,1(loss of plasmid)	0
34	31	0	<14
38	Not included in transmission analysis: not genome-sequenced		
39	34	2	Theatre (same day)
40	34	1	Theatre (same day)
43	34	1	<7
44	43	0	<21
49	44	0	0
50	Unknown		
51	Unknown		
52	Outbreak restarts after patient 52 nursed on bed previously used by patient 50		
53	Unknown		
54	Unknown		
55	54	0	Theatre (1 day gap)
57	50	0	<21
58	57	1	0
59	57	2	0
60	54	1 (loss of plasmid)	<14
61	57	2	Theatre (4 day gap)
62	57	2	0
63	62	1	0
64	57	0	<14
65	64	0	0

Early in the outbreak, epidemiological and genomic analyses indicated that transmission occurred primarily as a result of cross-infection between patients located on the same ward at the same time. Thus, all isolates from Genotypes 1.0 and 2.0 and most of the isolates from Genotype 4.0 came from patients who had stayed on the Ward 1. In some cases, long-term contamination of the ward environment was thought to account for transmission and this was confirmed by environmental swabbing in side rooms after patients had been discharged and the room cleaned (Table 1). For example, isolate E1 was recovered a day after patient 44 was discharged; genomic analyses revealed it shared the same SNV profile (Genotype 6.0) as four of the five MDR-Aci isolates from that patient. Similarly isolates E2-4 were taken a day after patient 55 was discharged and were found to show a one-SNV difference from a patient 55 isolate. In both cases, the patients suffered severe burns and each stayed in a single room for the entire hospital stay. Confirmation of contamination of the hospital environment led to a tightening of ward decontamination procedures.

Some outbreak strain acquisitions could not be explained simply by within-ward transmission, so we were forced to consider alternative routes of infection. As the outbreak progressed, we noticed that most of the affected patients made numerous visits to operating theatres: only five were never treated in an operating theatre. One particular theatre, specializing in the treatment of burns patients, was implicated in transmission between patient 34 (donor) and patients 40 and 39 (recipients). Consequently, in week 46 the burns theatre was closed and underwent deep cleaning (that is, decluttering of the operating theatre, followed by cleaning of all patient-associated equipment, non-fixed items, horizontal surfaces, walls, ceilings, ventilation shafts and storage areas with a chlorine-based disinfectant). Although there were several ward-based transmission events in the weeks that followed, no new theatre-acquired cases were observed for the subsequent 6 weeks and, for a time, the outbreak appeared to have ended.

Unfortunately, the outbreak resumed when a burns patient, patient 52, presented with an isolate from Genotype 6.0 in week 70. Initial epidemiological investigations failed to find any plausible direct ward- or theatre-based route of transmission that might link patient 52 with earlier outbreak cases. However, our finding of genotypic identity between the patient 52 isolate and previous outbreak isolates forced us to perform a more thorough epidemiological investigation, which uncovered a vehicle for transmission: patient 52 had occupied a specialised burns care bed that had been previously occupied by another Genotype 6.0 patient, patient 50. This prompted the development of a decontamination protocol for this specialised type of bed.

The outbreak spread to over a dozen new patients during the subsequent 9 weeks. Our suspicion once again focused on the burns theatre as the likely source of infection. This was confirmed when we obtained six isolates (E5-10) from environmental swabs of the burns operating theatre. All isolates from this phase of the outbreak, from patients and the environment, belonged to, or were closely related to, Genotypes 6.0 and 7.0. These findings prompted a second closure of the burns theatre, with deep cleaning in week 76. Following this second deep clean of the theatre the outbreak ceased and no further acquisitions of the strain were identified. The outbreak was formally declared closed in May 2013 when no inpatients were colonised or infected with the outbreak strain and there had been no new acquisitions for a period of 12 weeks.

## Discussion

Like many other hospitals, QEHB suffers from serial clonal outbreaks of MDR-Aci, which result from the importations of outbreak strains, often by military patients [7-9]. We have described a prolonged outbreak of MDR-Aci, in which bacterial WGS provided a powerful adjunct to conventional laboratory and epidemiological investigations. In so doing, we have built on our previous smaller-scale efforts on the genomic epidemiology of *A. baumannii* [7] and on the work of others on a polyclonal outbreak genome-sequenced using the 454 platform [28]. However, we have now benefited from the improved ease of use, cost-effectiveness, throughput and accuracy of the Illumina MiSeq platform. Unlike a recent epidemiological analysis of MDR-Aci in a US hospital [29]. We have focused on a single clonal outbreak and have used a whole-genome SNV-based analysis twinned with loss or gain of a plasmid to reconstruct strain evolution as the outbreak progressed.

We have shown that this particular pathogen, *Acinetobacter baumannii*, undergoes sufficiently rapid genome evolution within a hospital outbreak to allow SNV analyses to reveal modes and routes of transmission and distinguish between alternate transmission scenarios. When we combined evidence from over 30 genotypes with conventional epidemiological data, we were able to identify the sources of several unexplained transmission events and target additional investigations and infection-control interventions to reduce the risk of further transmission.

In this study, we were able to link patient-derived isolates directly to environmental isolates. Early in the outbreak, confirmation of contamination of the patients’ surroundings on the ward led to a tightening of ward disinfection procedures. Later, WGS showed that environmental isolates from a burns operating theatre were linked to patient isolates, which triggered closure of the theatre, followed by deep cleaning. Previous reports have highlighted that burns patients are particularly at risk of infection with *Acinetobacter baumannii* and that burns units are susceptible to outbreaks and have also implicated contaminated mattresses in infection [30,31].

We have demonstrated that there is considerable genotypic diversity within patient-associated and environmental populations of *Acinetobacter*. We discovered two examples of what were clearly mixed infections, where more than one species or strain of *Acinetobacter* was present in serial samples from the same site. In addition, when we genome-sequenced multiple isolates from multiple samples from a chronically infected patient, we found that the individual was carrying a cloud of variants of the outbreak strain, rather than a single SNV genotype. It remains unclear whether such genotypic diversity evolves *in situ* or whether multiple genotypes are acquired serially or en masse. Although some have suggested that such genotypic diversity precludes the reconstruction of transmission events from genomic data [32], the congruence and consistency we and others [12] see between genotypic and epidemiologic data in such analyses confirm the utility of such approaches. However, the presence of such genotypic diversity in pathogen populations challenges the long held practice of picking and propagating a single example of each colonial morphotype from a primary culture and suggests a role for metagenomic approaches that might capture bacterial genetic heterogeneity directly from the clinical sample without culture [33-35].

Although, in this study, WGS was initially largely retrospective, in the latter part of the outbreak, we were able to go from colony to SNV genotype in less than 1 week. Local WGS thus allowed us to rule patients and isolates in or out of the outbreak more quickly than through the reference laboratory. Excluding isolates meant that we could focus our efforts on determining the connections between genetically related cases, rather than trying to connect all cases of MDR-Aci.

## Conclusions

WGS is now poised to make an impact on hospital infection prevention and control, delivering cost-effective identification of routes of infection within a clinically relevant timeframe and allowing infection control teams to track, and even prevent, the spread of drug-resistant hospital pathogens.

## Abbreviations

MDR-Aci: Multi-drug-resistant *Acinetobacter*
MIC: Minimum inhibitory concentration
PFGE: Pulsed field gel electrophoresis
QEHB: Queen Elizabeth Hospital Birmingham
SNV: Single nucleotide variant
WGS: Whole-genome sequencing
